# IDSS: deformation invariant signatures for molecular shape comparison

**DOI:** 10.1186/1471-2105-10-157

**Published:** 2009-05-22

**Authors:** Yu-Shen Liu, Yi Fang, Karthik Ramani

**Affiliations:** 1 School of Mechanical Engineering, Purdue University, West Lafayette, IN, 47907, USA; 2 School of Electrical Computer Engineering (by courtesy), Purdue University, West Lafayette, IN, 47907, USA

## Abstract

**Background:**

Many molecules of interest are flexible and undergo significant shape deformation as part of their function, but most existing methods of molecular shape comparison (MSC) treat them as rigid bodies, which may lead to incorrect measure of the shape similarity of flexible molecules.

**Results:**

To address the issue we introduce a new shape descriptor, called Inner Distance Shape Signature (IDSS), for describing the 3D shapes of flexible molecules. The inner distance is defined as the length of the shortest path between landmark points within the molecular shape, and it reflects well the molecular structure and deformation without explicit decomposition. Our IDSS is stored as a histogram which is a probability distribution of inner distances between all sample point pairs on the molecular surface. We show that IDSS is insensitive to shape deformation of flexible molecules and more effective at capturing molecular structures than traditional shape descriptors. Our approach reduces the 3D shape comparison problem of flexible molecules to the comparison of IDSS histograms.

**Conclusion:**

The proposed algorithm is robust and does not require any prior knowledge of the flexible regions. We demonstrate the effectiveness of IDSS within a molecular search engine application for a benchmark containing abundant conformational changes of molecules. Such comparisons in several thousands per second can be carried out. The presented IDSS method can be considered as an alternative and complementary tool for the existing methods for rigid MSC. The binary executable program for Windows platform and database are available from .

## Background

Molecular shape comparison (MSC) has been playing an increasingly important role in computer aided molecular design, rational drug design, molecular docking and function prediction. The goal of MSC is to find the spatial properties common to two or more molecules. Especially in computer aided drug design, a critical problem of *virtual screening*, aimed at identifying the drug-like molecules likely to have beneficial biological properties, is comparing molecular shapes. An alternative virtual screening technique consists of searching a molecular database for compounds that most closely resemble a given query molecule [1-4]. The underlying assumption is that the molecules similar to the active query molecule are likely to share similar properties. This similarity can be in terms of molecular geometrical shapes or descriptors. A number of previous studies have concerned shape comparison of molecules [1,2,5-10]. Most existing MSC methods are only effective for comparing 3D rigid objects, but they can not handle the deformed shapes of flexible objects well. Nevertheless, many molecules of interest are flexible and undergo significant shape deformation as part of their function. When flexible molecules in different conformations are compared to each other as rigid bodies, strong shape similarities might be missed. To address the issue we developed a new method for comparing molecular shapes, which is insensitive to molecular shape deformation compared to previously rigid methods.

### Methods of molecular shape comparison

The molecular shape has been widely acknowledged as a key factor for biological activity and it is directly related to the design of selective ligands for protein and DNA binding. To exploit the shape similarity of molecules in the shape-based molecular design, a useful tool is MSC that compares the shapes of two or more molecules and identifies common spatial features [11,12]. Such comparison can lead to some alternative models in the process of drug design. An additional advantage of MSC is that no specification of chemical structure is made and therefore the molecules with shape similarity, but with different chemical structure, can be found [1,2]. However, the efficient MSC is currently a challenge [1,2,11,12] due to the high complexity of 3D molecular shapes.

Ballester et al. [1,2] divided the MSC methods into two categories: *superposition *and *descriptor *(or *signature*) methods. The former relies on finding an optimal superposition of molecules, and the later (i.e. *non-superposition*) is independent of molecular orientation and position.

#### Superposition MSC

The superposition methods are a popular family of MSC methods based on the optimal superposition/alignment of two or more molecules. The early superposition method was developed by Meyer and Richards [13] to measure the similarity of molecular shape. Masek et al. [14] compared molecular shapes by optimizing the intersection of molecular surfaces. ROCS (Rapid Overlay of Chemical Structures) is an available superposition method [15] and it performs shape-based overlays of two molecules by a local optimization process. The algorithm is based on the earlier implementations of molecular shape comparison described by Masek et al. [16], which quickly finds and quantifies the maximum overlap of the volume of two molecules [11,12]. Rush et al. [17] described a shape-based 3D scaffold hopping method, which is an application of ROCS to a bacterial protein-protein interaction. Recently, Natarajan et al. [18] compared rigid components of molecules by segmenting their surfaces based on Morse theory. The superposition MSC methods require a priori superposition/alignment of molecular shapes into a coordinate system, which is difficult to achieve robustly. The reader may consult Refs. [1,2] for a review of many available methods of superposition MSC.

#### Descriptor/signature MSC

Another category of shape comparison methods uses descriptor/signature to represent the shape of molecule. The kind of methods is non-superposition that computes the similarity score by comparing the corresponding descriptors between two molecular shapes. A 3D shape descriptor, or called signature, is a compact representation for some essence of the shape. The shape descriptor is usually used as an index in a database of shapes and enables fast queries and retrieval. The descriptor methods are simpler than the traditional superposition methods that require shape superposition/alignment, feature correspondence, or model fitting [6,7]. An early molecular shape description is developed by Bemis et al. [19] by considering each molecule as a collection of its 3-atom submolecules. Nilakantan et al. [20] also introduced a method for the rapid quantitative shape match between two molecules or a molecule and a template, using atom triplets as descriptors.

Several recent works related to molecular shape comparison using shape descriptors have been developed including shape distribution descriptor, spherical harmonic signature, 3D Zernike descriptor, etc [3,5-8,21-25]. These descriptors are rigid-body-transformation invariant, and they are effective for matching rigid objects. Nevertheless, none of these methods is deformation invariant and they can not support flexible molecular shape comparison.

Deformation invariant representation of nonrigid or flexible shape like articulated objects is a challenging problem in the field of shape analysis. Several recent works focus on this problem [26-30]. One class of approaches focuses on topology or graph comparison for determining the deformation [27], but the graph extraction process is often very sensitive to local shape changes. Furthermore, graph comparison cost increases proportionally with the graph size, resulting in relatively slow comparison and retrieval times. In [26], Elad and Kimmel presented a bending invariant representation for a pitch of surface based on multidimensional scaling, but the geodesic distance is sensitive to shape changing [31] and therefore it is not appropriate for protein comparison. Jain et al. [28] presented a spectral approach to shape-based retrieval of deformation 3D models, but this method is not appropriate for protein models with many holes. Recently, Gal et al. [29] proposed the local diameter shape signature by computing the distance from surface to medial axis. Other methods take into account local features on the boundary surface of the shape in the neighborhood of points [30]. Usually, these local techniques are based on matching local descriptors. However, many times they do not perform well on global shape matching because of their local nature they do not provide a good signature of the overall shape [29]. These existing descriptors can not perform well for flexible molecules due to their complex shape deformation.

### Distance signatures

In 3D shape retrieval, the simplest and most widely used shape signatures is the distance signature between sampling point pairs on shape surfaces. Our work also belongs to this category. We introduce three representative distance signatures: *Euclidean distance (ED)*, *geodesic distance (GD) *and *inner distance (ID)*. The ED signature [7] usually is represented by a histogram of distance values and it is formed by three steps: 1) sampling uniformly random points from the shape surface, 2) computing the ED between the sampled point pairs, and 3) building the histogram of corresponding distance values. After finishing computation of the signature histogram, the similarity scores of shapes are defined as the distance between their histograms. A histogram is actually a one-dimensional vector. However, the ED histogram between pair of points on a shape surface is sensitive to shape deformation. An alternative distance signature is to replace ED by geodesic distance (GD) [26]. The GD between any pair of points on a surface is defined as the length of the shortest path on the surface between them. Since the GD is invariant to surface bending, the stretched surface forms a bending invariant signature of the original surface. Although the GD is insensitive to surface stretch, it is sensitive to shape deformation, as shown by [31]. The GD does not work well for our purpose.

The work most related to ours is [31], in which a novel 2D inner distance measurement is presented for building 2D shape signatures. The ID signature is robust to articulated deformation and it is more effective at capturing shape structures than both ED and GD. In 2D case, the ID is defined as the length of the shortest path between landmark points within the 2D silhouette. However, no algorithm for ID computation of 3D shapes is given due to the complexity of 3D shapes so far.

## Results

### Overview of approach

**Figure 1:**
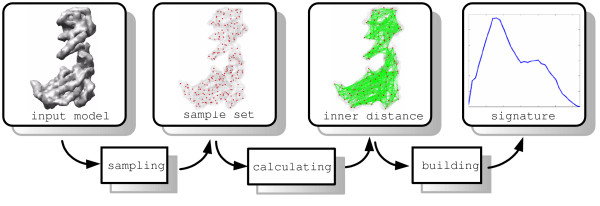
**Flowchart of IDSS**. Given a molecular shape, three independent steps contain sampling (red points), calculating the inner distance (green line segments) between all sample point pairs, and building the signature (blue histogram). Here the input shape is the volumetric data with the simulated 8 Å resolution density map for GroEL (PDB code: 1aon).

**Figure 2:**
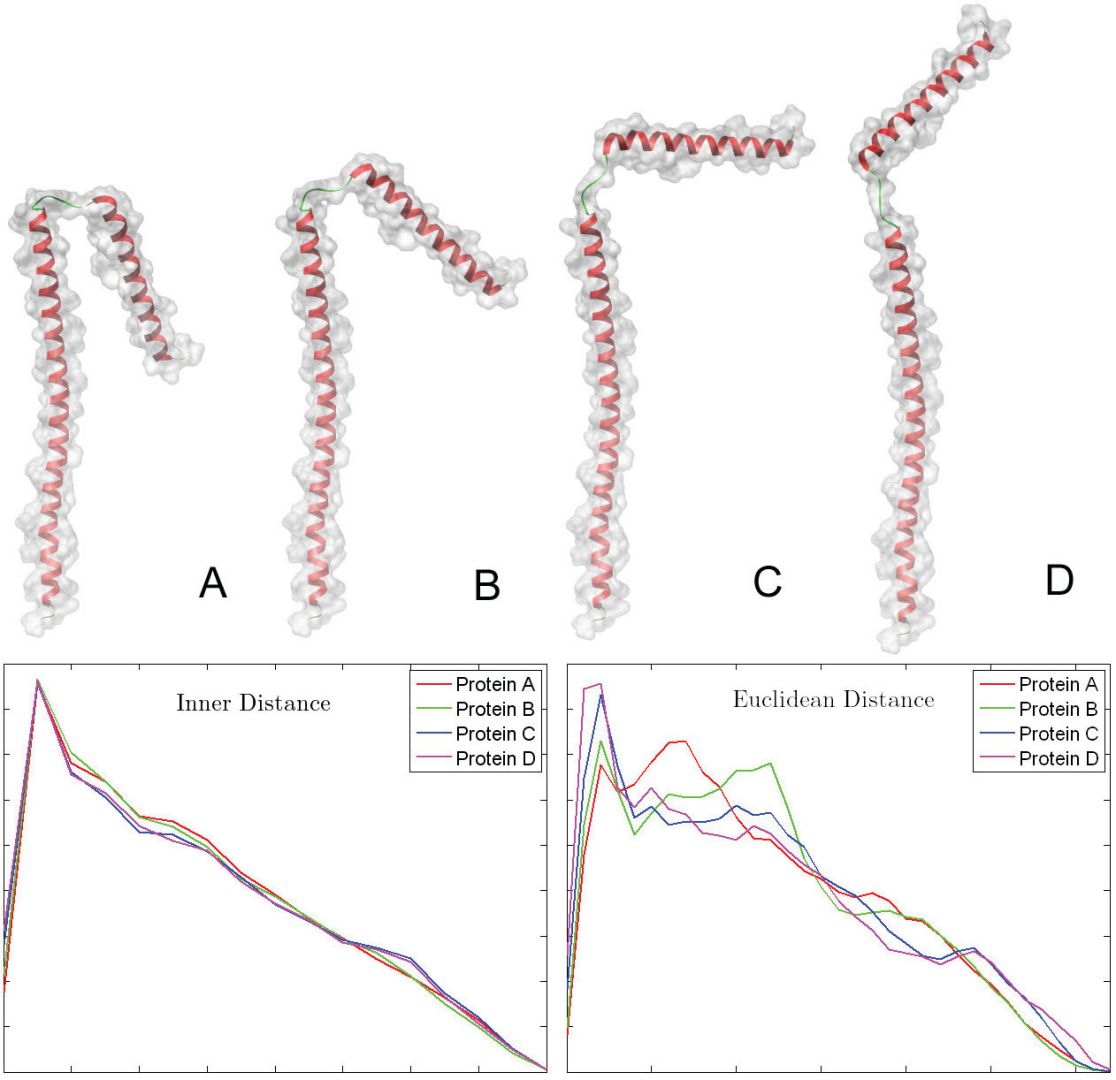
**Our inner distance (ID) signature is compared, for instance, to Euclidean distance (ED) signature from **[7]. The first row shows the input four artificial proteins with the same main chain orientation but with different molecular shapes. The second row shows the ID and ED signatures. In each plot, the vertical axis represents distance distribution. Note that ID is not sensitive to shape deformation, so four signatures are almost consistent; in contrast, ED is strongly sensitive to deformation.

Here we introduce a new technique, called Inner Distance Shape Signature (IDSS), for describing the 3D shapes of flexible molecules. Our work can be regarded as an extension of inner distance from 2D to 3D for computing the deformation invariant shape signatures of flexible molecules. The procedure of computing the IDSS of molecular is given as follows. First, we obtain a set of points sampled uniformly from a molecular surface using Lloyd's algorithm of k-means clustering. Then a new algorithm is presented for checking the *inside visibility *between sample point pairs; based on their inside visibility, we define a graph and compute the inner distances using a shortest path algorithm in the graph. Finally, we build a signature of inner distances for measuring the global geometric properties of the molecule. The core procedure can be divided into three steps: sampling, calculating inner distance and building signatures (see Figure 1). These techniques have been implemented in a software package called the IDSS program. Figure 2 illustrates the comparison between IDSS and rigid methods. The source molecule comes from *Drosophila sp*. (PDB code 2spc, chain A), where 2spcA has one long helix and one short helix. The four artificial molecules (A, B, C and D in Figure 2) are formed by fixing the long helix of 2spcA and rotating the short helix about 10, 45, 90 and 120 degrees round *x*-axis, respectively. The input four artificial molecules have the same main chain orientation but with different surface shapes, where the IDSS is computed with surface shapes of molecules. Note that our inner distance signatures remain largely consistent for the four deformed molecular shapes of the same protein, while the previous rigid descriptor [7] is strongly sensitive to shape deformation.

### Definition of inner distance

First, we extend the definition of inner distance (ID) in 2D objects [31] to 3D shapes. Let *O *be a 3D shape as a connected and closed subset of ℝ^3^. We denote the boundary surface of *O *by ∂*O*. Given two points **x**, **y **∈ ∂*O*, the ID between **x **and **y**, denoted as *d*(**x**, **y**; *O*), is defined as the length of the shortest path connecting **x **and **y **within *O*.

**Figure 3:**
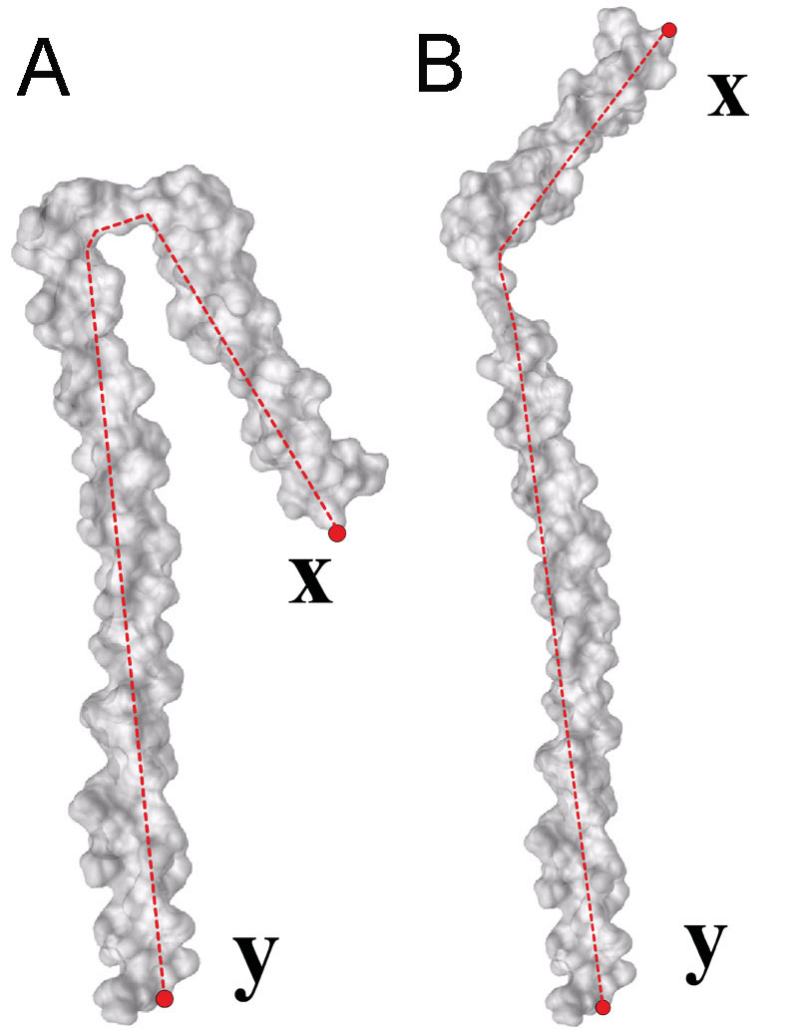
**Illustration of definition of the inner distance**. The red dashed lines denote shortest paths within the shape boundary surface that connect two landmark points **x **and **y**. The right object B is articulated deformation to the left one A, and the relative change of the inner distances between the corresponding pair of points (e.g. **x **and **y**) during articulated deformation are small.

Figure 3 gives the illustration of the ID definition, where the red dashed lines denote the ID paths between two landmark points **x **and **y**. Note that the object B is an articulated deformation of the object A. In contrast, the Euclidean distances (ED), defined as the length of the line segment between two landmark points (**x **and **y**), does not consider whether the line segment crosses the shape boundaries. Intuitively, this example shows that the ID is insensitive to articulated deformation, while the ED does not have this property. The significant advantage of ID is that it reflects shape structure and articulated deformation without explicitly decomposing the shape into parts. Note that there may be multiple shortest paths in rare cases, and we arbitrarily choose one. We are interested in 3D shapes defined by their boundaries, hence only boundary points are used as sampling points. ID reduces to ED when *O *is convex.

#### Articulated deformation and hinge-bending movement

Ling and Jacobs [31] have proven that the ID is insensitive to the shape articulated deformation by decomposing the shape into some rigid parts connected by junctions. An articulated shape *O *is described with the following conditions: 1) *O *can be decomposed into several *parts *that are connected by *junctions *(or hinge); 2) the junctions between parts are very small compared to the parts they connect; 3) the articulation of *O *as a transformation is rigid when limited to any part but can be non-rigid at the junctions. The relative ID change is very small for the articulated objects, so ID is insensitive to articulations. Molecules are flexible and can be regarded as an articulated shape. Many molecules contain flexible structures such as loops and hinge domains. Some recent studies demonstrated that the activity of many molecules induces conformational transitions by *hinge-bending*, which involves the movement of relatively rigid parts of a molecule about flexible joints [32-34]. In hinge-bending, parts of the molecule rotate with respect to each other as relatively rigid bodies, on a common hinge. The hinge-bending of molecules can be treated as a special shape articulated deformation. In Figure 2, each molecule contains two domains (two red helices) that are rigid regions and also contains one hinge (green loop) that is a flexible region.

### Data set of molecules

A molecule is represented by a set of overlapping spherical atoms. The exposed surface of these spheres represents a molecular surface that defines the boundary of a single molecule' volume. In this paper, we consider the input data as a volumetric/voxelized representation of molecular shape. There have been numerous works on this representation, such as for binding sites determination [35], molecular shape comparison [8], the cryo-electron microscopy (cryo-EM) data [36,37], and 3D shape searching [38]. We consider a volumetric model as a uniform 3D lattice consisting of object points *O *and background points . We represent the 3 × 3 × 3 neighborhood of each lattice point **x **by *N*(**x**), which is a set of 26 points and each point (other than **x**) that share a common grid edge, face, and cell with **x**. The boundary surface of *O *is defined as

(1)

**Figure 4:**
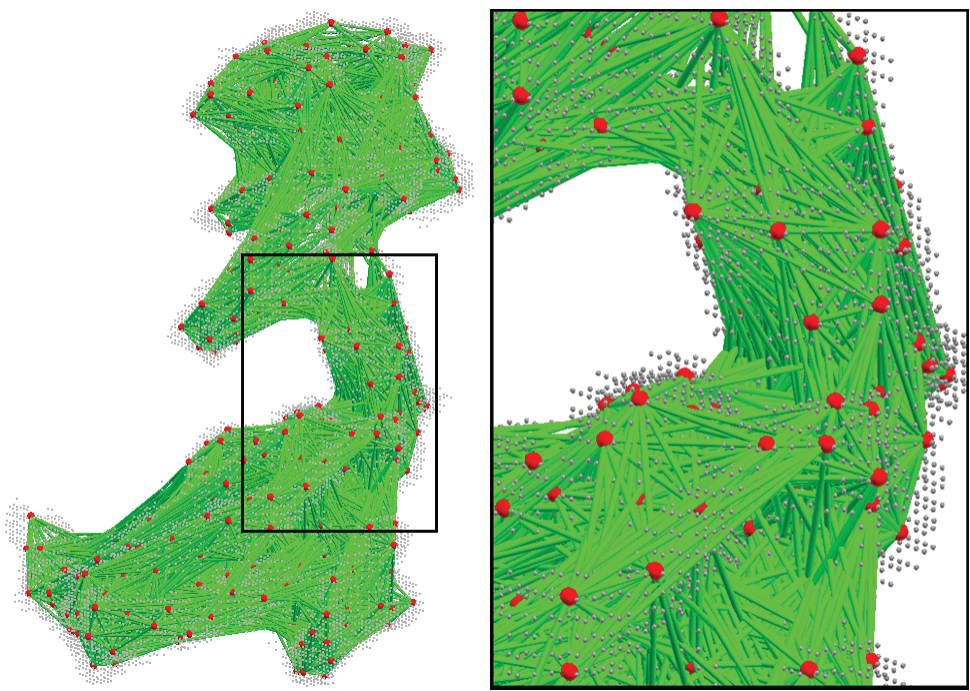
**Illustration of computing the inner distance for the protein (PDB code: **1aon). All boundary points of the shape are colored in light gray. Left, the shape with 500 uniform sample points (red color) and their inner distance (greed color). Right, a detail of the middle of the graph. Note that the inner distances capture the shape.

Figure 4 shows all boundary points of ∂*O *colored in light gray.

In the preprocessing stage, the molecular shape is built. The molecular shape in the MRC file format is directly used for our program as the default input. The MRC volumetric data can be generated by using a way described by [8]. First, the MSROLL [39] program in Molecular Surface Package is used to compute the Connolly surface (triangle mesh) of the molecule using default parameters. Next, the triangle mesh is placed in a 3D cubic grid of *n*^3 ^(such as *n *= 64), compactly fitting a molecule to the grid. Each lattice point is assigned either 1 or 0; 1 for object points *O *and 0 for background points . Alternatively, the users may use some commercial softwares for getting the MRC files of molecular models, such as Chimera [40] and EMAN [37].

We have implemented the technique presented in the previous section and tested it on a set of molecules. The algorithm described above is implemented in C++. To show the ability of the IDSS approximating the molecular shapes, we first select a couple of complicated examples for visualizing their IDSS. To demonstrate the utility of deformation invariant signatures, we develop a shape search system of flexible molecules and test this system for a benchmark containing abundant conformational changes of molecules.

### Examples of simulated data

**Figure 5:**
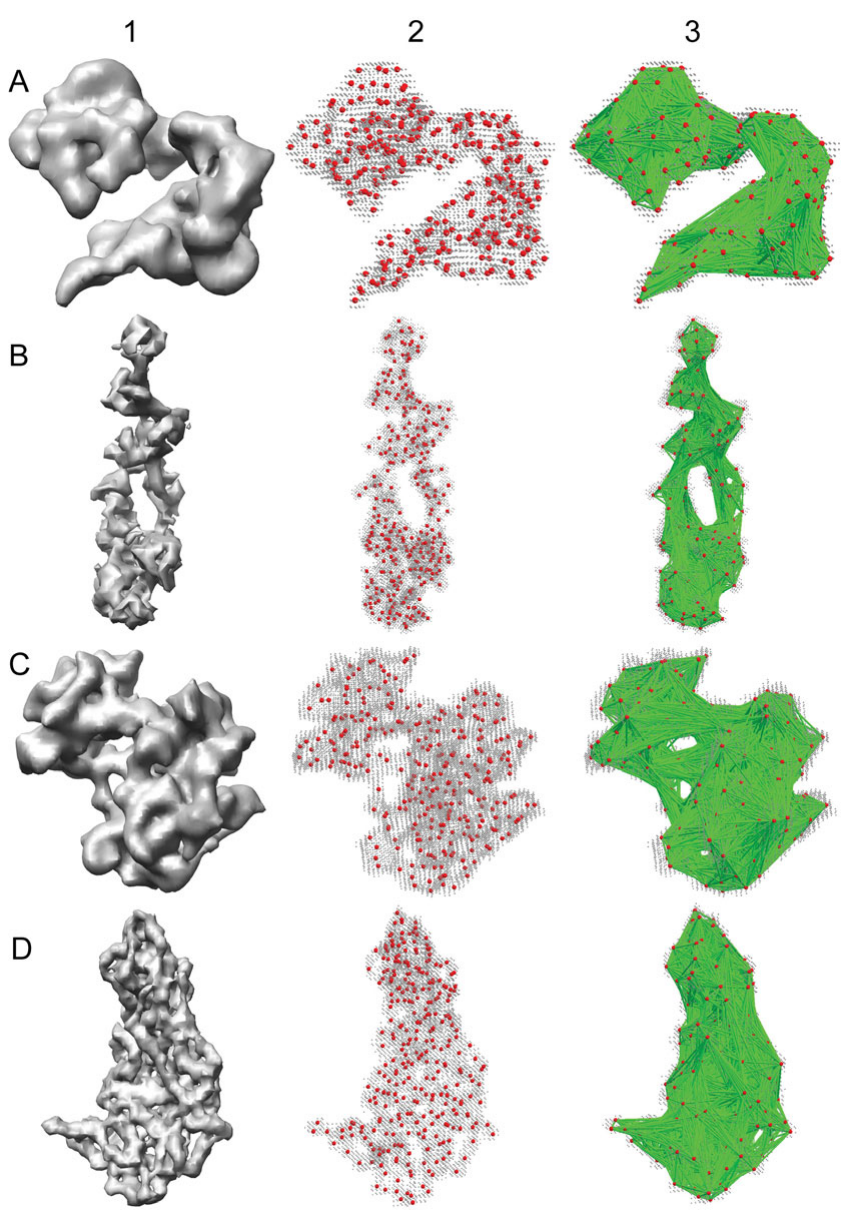
**(A-D) test four protein models with 500 sample points each**. (A) 1ctr. (B) 1b7t. (C) 1irk. (D) 2btv. Column 1 shows the isosurfaces with the simulated 8 Å resolution density maps for the four models. Column 2 shows the uniform sample points, while column 3 shows the pathes of inner distances. Points on boundary surfaces are colored in gray, sample points are colored in red, and the edges of inner distance are colored in green.

**Figure 6:**
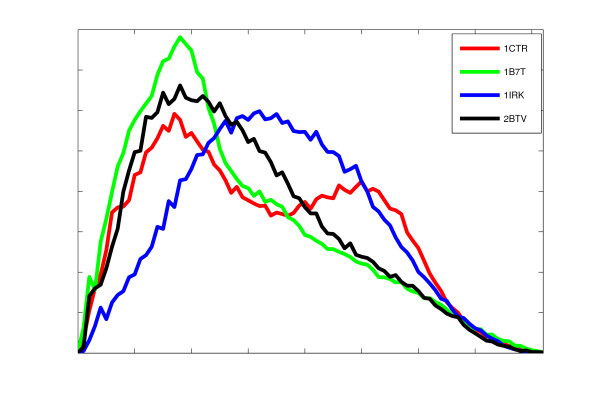
**The inner distance signatures of four models are given**.

The ability of inner distance to represent deformation invariant shape signatures of flexible molecules is first tested on some unrelated proteins (PDB code: 1ctr, 1b7t, 1irk and 2btv). Previously, these structures are used in the assessment of structure recognition in cryo-EM [41]. Four protein models with the simulated 8 Å resolution density maps are shown in Figure 5. The computed inner distances approximate well the global shapes of four proteins. Note how the inner distances capture the holes for 1b7t and 1irk. This apparent differences of the global surface shapes are also reflected by distinctive inner distance signatures shown in Figure 6.

#### Articulated deformation insensitivity

As described above in this paper, the most attractive one of advantages of IDSS is deformation insensitivity for 3D articulated shapes in contrast with the traditional rigid descriptors. In addition, the IDSS also captures some global geometric properties which are scale, translation and rotation invariant. However, in practice the IDSSs of deformation shapes of one same protein are not exactly identical. This error is caused by two reasons. One is that the molecular surface shape *O *is discretized into the volumetric format, where *m *sample points on the boundary surface ∂*O *of *O *are only used for approximating the global inner distances. Smaller *m *does not sufficiently approximate ∂*O*, while larger *m *requires more computation time and space. In our implementation, we typically choose *m *= 500 for both a small approximation error and little computation time.

**Figure 7:**
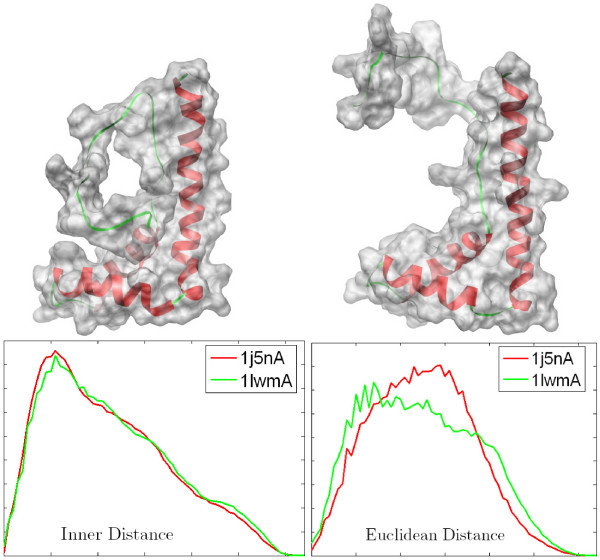
**The ID signature compared, for instance, to EDsignature**. The first row shows the input two conformations 1j5nA (left) and 1lwmA (right) of the same protein. The second row shows the ID and ED signatures. Note that ID is not sensitive to shape deformation, so two signatures are very close; in contrast, ED is strongly sensitive to deformation.

The second reason is that the size of the loop and hinge regions of deformations affects the IDSS computation. Intuitively, for smaller the loop and hinge change compared to the overall size of the molecular shape, the inner distance changes are smaller. Figure 7 shows an example of articulated deformation insensitivity of the IDSS. Here, the two molecules used are two conformations of the same protein (PDB code: 1j5nA and 1lwmA), and the relative change of the loop (green) on the left top are large. This results in some errors in the IDSS, but the two IDSSs are still very close with the similar histogram. In contrast, the traditional rigid descriptors fail in deformation detection (see the Euclidean distance signature in this figure).

### A search system of flexible molecules

To assess the efficacy of the proposed signature, we have incorporated the new method into a system of molecular shape comparison. We have chosen to test our method on a benchmark set of molecules found in the Database of Macromolecular Movements (MolMovDB) [42]. MolMovDB presents a diverse set of molecules that display large conformational changes in proteins and other macromolecules, which can be found at: . The benchmark data set is classified 214 groups with the total 2,695 PDB files, where each is named the corresponding group ID. The number of conformations in different group may be different. This benchmark has been used in predicting protein structures and hinge predictor [32,43]. The developed search system of flexible molecules provides a tool with which users can retrieve molecules from the benchmark based on their shape attributes. In our current program, the user selects a query molecule from the database and the program computes the similarity scores for all molecules in the database using the methods described in this paper. The program then shows the query molecule and the similar molecules in the database.

**Figure 8:**
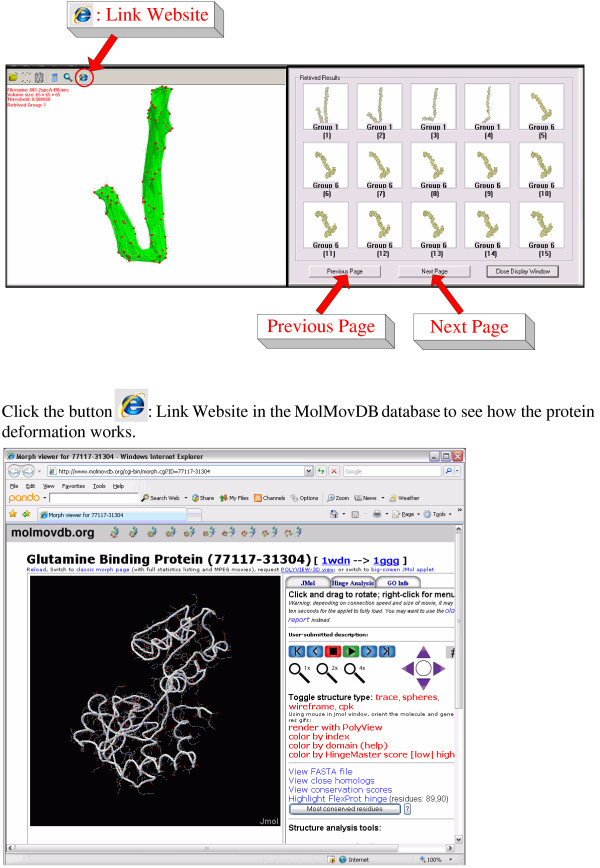
**A screen shot from flexible molecular shape comparison system**.

Figure 8 shows the framework and its visual appearance. In the interface of our program, the query molecular is displayed on the left and the retrieved results including Group ID are shown in the right dialog box. The example in Figure 8 shows a query molecule from Group 1 and some retrieved molecules. Especially, in our database, there are only four molecules in Group 1, our method can totally find them in the first four retrieved results although they have different deformations. Note that the current page in the retrieved results only shows the most related 15 results. To see more results, the users can click the button "**Next Page**" in the dialog box and the other groups will come in the next page. Here, we renamed each group "Group + an unique number". If the users want to learn more details of the query protein, they can click the button "**Link Website**" in our program to connect the corresponding website in the MolMovDB database to see how the protein deformation works.

In the MolMovDB benchmark we have pre-calculated all inner distance signatures of queries on the database and display molecules using images in the dialog box of retrieved results. The inner distance signatures allow rapid search on the system because a molecular shape is compactly represented by a 1D vector. If a query molecule is already transformed into the inner distance signature, a search to the current benchmark data set takes less than half a second.

### Comparison with existing methods

**Figure 9:**
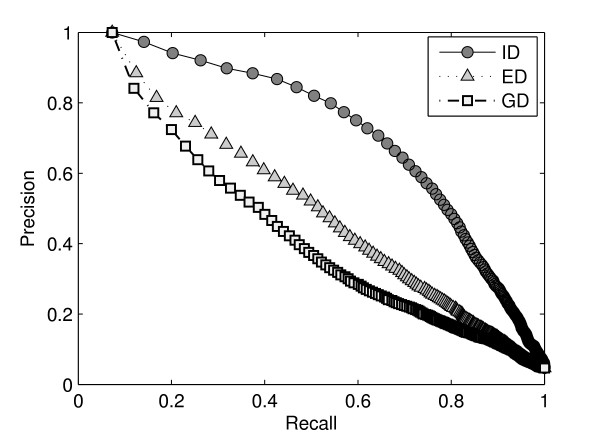
**Precision-recall curves computed for three distance signatures: ID, ED and GD for the MolMovDB database**.

The ID shape signature is first compared with two other distance signatures: Euclidean distance (ED) and geodesic distance (GD) in terms of the performance on retrieving similar molecular structures. We use standard evaluation procedures from information retrieval, namely *precision-recall *curves, for evaluating the various shape distance signatures [44]. Precision-recall (PR) curves describe the relationship between precision and recall for an information retrieval method. Precision is the ratio of the relevant models retrieved to the retrieval size. Recall is the fraction of the relevant models retrieved for a given retrieval size. A perfect retrieval retrieves all relevant models consistently at each recall level, producing a horizontal line at precision = 1.0. However, in practice, precision decreases with increasing recall. The closer a PR curve tends to the horizontal line at precision = 1.0, the better the information retrieval method. Figure 9 shows the PR curves of three distance signatures for the MolMovDB database. The results show that the ID method performs better than ED and GD at average level for flexible molecules. As we discussed previously, although the GD is insensitive to surface stretch, it is sensitive to 3D shape deformation [31]. GD is sensitive to 3D shape deformation. From our experiments we also found that some molecules with one domain are often judged as similarity to some ones with two or three domains when using GD signatures. In the MolMovDB database, GD can not give good searching results as well as ED. Furthermore, we compared our signatures with three known rigid descriptors: the spherical Harmonic descriptor, the solid angle histogram and the eigen value model [44]. The all three methods have been developed and used for searching of rigid shapes in computer graphics, engineering domain, and molecular shape comparison. One recent work [8] has compared the differences between shape descriptors with the cleaned SCOP protein classification database. In their paper, the 3D Zernike descriptor retrieved the better results than the above rigid methods based on the consistency of the rigid shapes. However, protein are flexible molecules that undergo significant structural changes and shape deformations as part of their function, and the existing rigid descriptors all fail on deformation detection (e.g. for examples in Figure 2 and Figure 7).

## Discussion

In this section, we present several potential applications for IDSS by replacing the conventional rigid shape descriptors in molecular shape comparison and also discuss some limitations of our approach.

### Searching molecular databases for drug design

In rational drug design, a unifying principle is the use of either shape similarity or complementarity to identify compounds expected to be active against a given target [1-3,12]. Shape similarity is the underlying foundation of ligand-based methods that seek compounds with structure similar to known actives. Shape complementarity is the basis of most receptor-based design methods, which identify compounds complementary in shape to a given receptor. One of the future works is to apply the IDSS method to some large and diverse molecular databases for both ligand- and receptor-based molecular design.

There are some methods that have focused on searching diverse molecular databases based on the descriptor MSC methods. For instance, Nilakantan et al. [20] searched the ten molecules with the highest shape similarity score in a database consisted of 22,495 compounds derived from the Cambridge Crystal File. Their technique can be used to screen large databases to eliminate those candidates which have a low shape similarity with the template. Hahn [45] described a three-phase database searching strategy for rapidly finding compounds similar in shape to a given shape query. This used database contained 45,579 compounds and 1,949,459 total conformations. Zauhar et al. [3] tested their shape signature method to the Tripos fragment database and the NCI database (113,331 compounds) under two different metrics. Recently, Ballester et al. [1,2] presented ultrafast shape recognition to search several compound databases for similar molecular shapes. The tested databases include the Vendor Database (2,433,493 commercially available compounds) and an independent benchmark from DrugBank. Our IDSS may replace the existing shape descriptors used in the above molecular databases. Searching molecular databases for drug design will be the subject of a separate publication.

### Protein structure retrieval

With the rapidly increasing number of known protein structure data, fast structural comparisons and retrieval methods are necessary to protein structure databases. Many structural comparison methods of proteins have been proposed for computing the similarity scores, and most of them are based on protein structure alignment, such as DALI [46] and CE [47]. Structural alignment aims to compare a pair of structures, where the alignment between equivalent residues is not given prior. Therefore, an optimal sequence alignment needs to be identified, which has been shown to be NP-complete [48]. In addition, several methods consider the hinge regions for aligning the protein rigid subparts [33,49]. Recently, we also presented a structural comparison method for flexible proteins using least median of squares [50]. The reader may consult [51] for comprehensive evaluation of protein structure alignment methods.

Our IDSS method can be used as a search for similar protein structures. One main advantage is that the shape-based protein searching method does not produce an alignment between two proteins (i.e. correspondence between amino acids). The standard benchmark data sets used to demonstrate the effectiveness of a similarity search are SCOP and CATH at various homology thresholds. It is expected that the presented IDSS method can be considered as an alternative and complementary tool for the existing methods for protein structure comparison and rigid molecular shape comparison.

### Discovery of high resolution structural homologues from cryo-EM maps

Computer reconstruction of cryo-EM images approximates the overall shape and topology of 3D volumetric object of macromolecular complexes [41,52-54], where it is not a trivial task to determine the structure information due to the low resolution. The obtained cryo-EM data is a 3D grid, called cryo-EM map, in which every voxel is assigned a density value. Only the overall shape and possible component boundaries are visible at low resolution; individual components become apparent at intermediate resolution. Many works have been presented for fitting high resolution structures of individual subunits into a cryo-EM map of a protein complex. Lasker et al. [54] divided the different approaches into two categories. One class of approaches assumes that the input is a cryo-EM map of a complex and an atomic resolution structure of one of its components, and the aim is to fit the given component into its location in the cryo-EM map. In many cases, only the cryo-EM map is available, whereas the atomic structures of individual components in a complex are unknown. Another class of approaches looks for closely related atomic structures of the complex's components and fits them into the map, which is a challenge. The previous methods search for structural homologues of the complex's domains based on sequence alignment or correlation scores, and then fit them into the map. To align atomic resolution subunits into cryo-EM maps, EMatch method [54] first identifies helices in an input cryo-EM map. It then uses the spatial arrangements of the helices to query a data set of high resolution folds and finds structures that can be aligned into the cryo-EM map. One key step in EMatch is to detect helices in cryo-EM. However, identification of secondary structure elements in low or intermediate resolution density maps still is a difficult open problem [41]. In addition, Baker et al. [41] discussed a framework for simultaneous identification of both *α *helices and *β *sheets in intermediate resolution density maps.

In the spirit similar to searching a data set used in EMatch, one possible solution is to first convert all proteins of the database into the density maps in the same resolution. Then we may search the converted database of protein surfaces for compounds that most closely resemble the input query cryo-EM. One main advantage of the strategy is to avoiding detecting *α *helices and *β *sheets for the input croy-EM. Most existing rigid MSC methods can work on the above searching step. Our IDSS method can also be directly used for searching for the most related proteins of the complex's components as an alternative method by considering the deformation of flexible proteins.

### Combining other characteristics into the signature

The IDSS algorithm presented in this paper belongs on molecular shape comparison. The current implementations only take advantage of geometry information of molecular shapes without chemical features. However, in many applications, such as matching in protein-protein or protein-ligand (drug) docking/design, chemistry is also very useful [55]. In fact, our current signature can be directly combined with some chemistry information. Specifically, other characteristics of a molecular surface, such as electrostatic potentials, might be naturally incorporated into the inner distance signature by considering a high dimensional sample point coordinate. For example, a molecular boundary surface ∂*O *in Eq. 1 can also be described as a set of 4D points ∂*O *= {**p**_*i *_= (*x*_*i*_, *y*_*i*_, *z*_*i*_, *c*_*i*_)}, where *x*_*i*_, *y*_*i*_, and *z*_*i *_are three geometry coordinates of the sample point **p**_*i *_and *c*_*i *_denotes its value of charge. The inner distance can be computed as the length of the shortest path between four-dimensional points. In the future, we intend to consider adding other chemical features into our signature.

### Limitation

**Figure 10:**
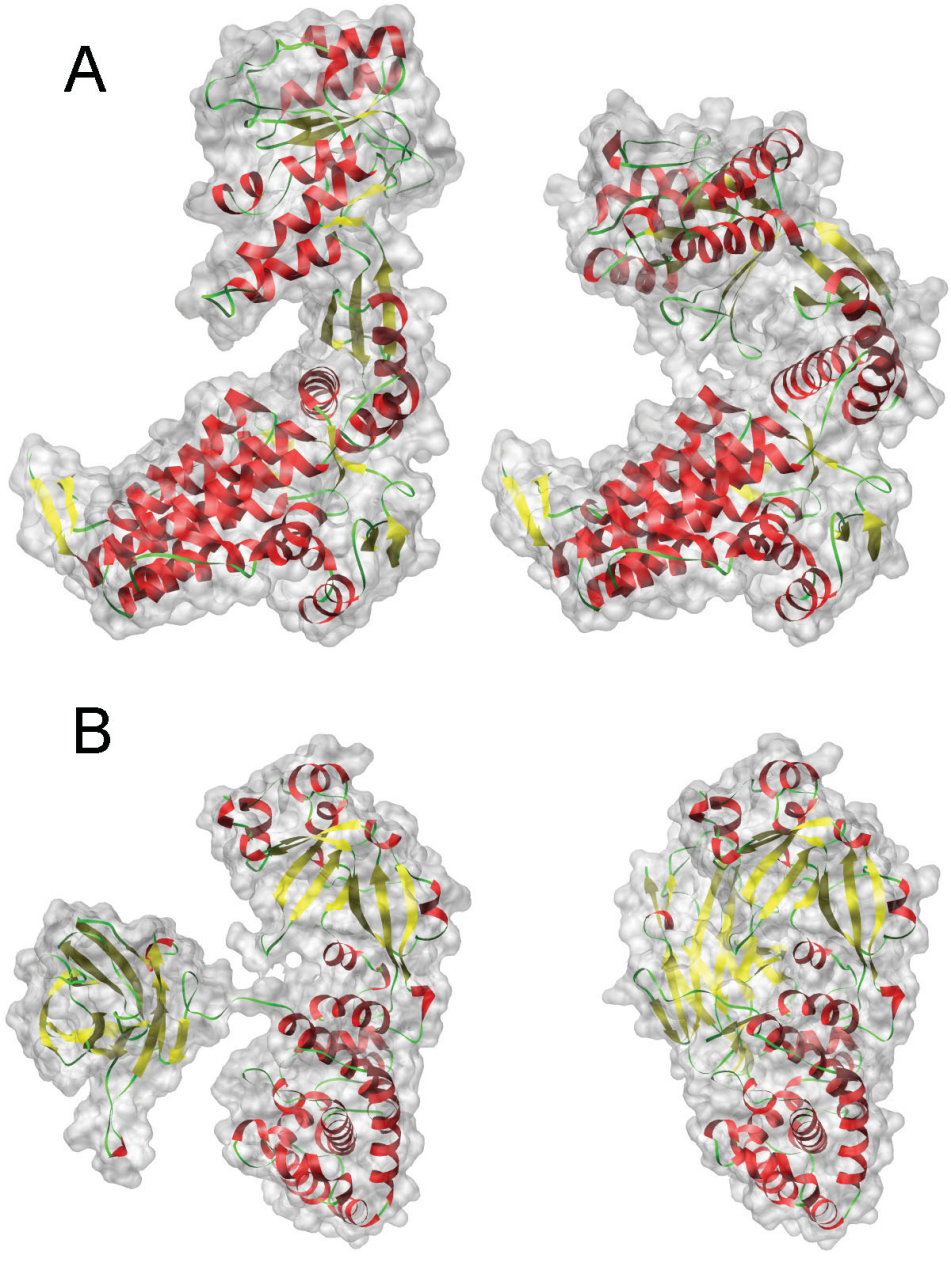
**Examples of protein conformation pairs but with very different shape structures**. (A) shows GroEL: 1kp8 (left) and 1aon (right), where 1kp8 has two separate domains and the corresponding domains of 1aon touch together. (B) shows Diptheria Toxin: 1ddt (left) and 1mdt (right), where 1ddt has several separate domains and the corresponding domains of 1mdt touch together.

A limitation of our approach is that the calculation of the ID is sensitive to the topology changes in shape. Figure 10 shows two examples of protein conformation pairs but with different topology structures. In Figure 10(A), two molecules are two conformations of GroEL (PDB code: 1kp8 and 1aon), where the intermediate domain of 1kp8 swings down towards the equatorial domain and the central channel so that the surfaces of two domains intersect in 1aon. In Figure 10(B), two molecules are two conformations of Diptheria Toxin (PDB code: 1ddt and 1mdt), where 1ddt has several domains but 1mdt shrinks together. The inner distance signatures will be very different between conformations in the term of the shape topology changes. However, the special cases with shape topology changes are not very usual in protein deformations. Our method can work well for most molecular shape deformation without topology changes. In many ways the definition of a signature which is both effective and highly robust to the object representation remains a challenge [29].

**Figure 11:**
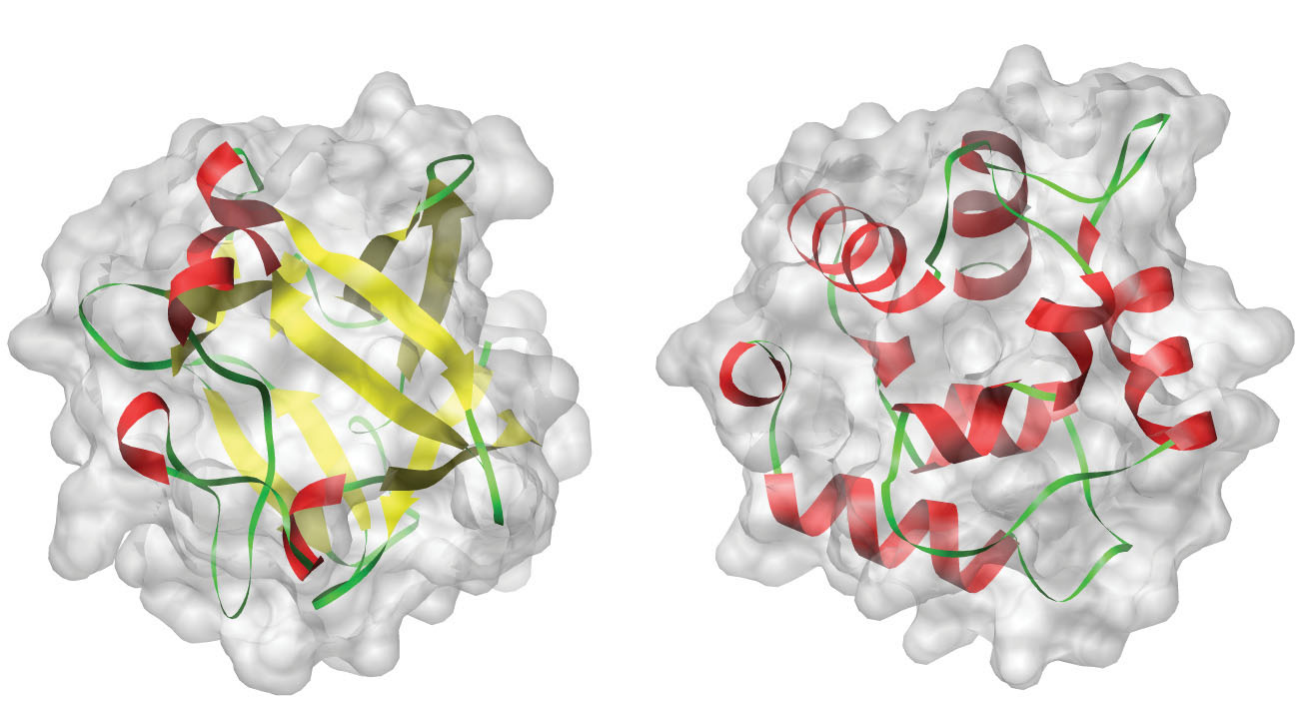
**Examples of proteins: **1barA**(left) and **1rro**(right) have similar geometry shapes but with different main chain orientation **[9].

Another limitation of molecular shape comparison is that the shapes with similar descriptors perhaps have no evolutionary relationship. Figure 11 shows a pair of proteins 1barA and 1rro, which is provided in Ref. [9]. The two proteins have very similar geometry shape descriptors, but they have very different main chain orientation. Most molecular comparison methods based on their shapes are not available for distinguishing the special cases. In particular, IDSS can be combined with classical structure alignment algorithms for protein shape retrieval. For example, IDSS first can be used to retrieve an initial small subset for a query protein, and then some conventional structure comparison methods, such as CE and DALI, can compute main-chain similarity in the small subset.

## Conclusion

A new method for molecular shape comparison (MSC), called IDSS (Inner Distance Shape Signature), has been presented. IDSS does not require previous alignment of the molecules being compared. We show that the IDSS is deformation insensitive and is good for approximating the complicated shapes of flexible molecules. In contrast, most existing MSC methods are effective for only comparing rigid objects and they can not handle shape deformation of flexible objects well. We have evaluated and demonstrated the effectiveness of IDSS within a molecular search engine application for a benchmark on MolMovDB. The new signature achieves good performance and retrieval results for different classes of flexible molecules with the efficiency of comparing histogram signatures. The presented IDSS method can also be applied to the molecular surface representation, such as the Connolly surface, by verifying whether a segment is inside the molecular surface. Moreover, we also showed several potential applications for IDSS by replacing the conventional rigid shape descriptors in molecular shape comparison, including searching molecular databases for drug design and protein structure retrieval.

## Methods

The IDSS algorithm for computing the inner distance shape signature of a object *O *is given at Algorithm 1:

### Algorithm 1 (IDSS)

1. Sample uniformly *m *points *S *= {**p**_1_,...,**p**_*m*_} on the boundary surface ∂*O *of *O *using Lloyd's algorithm of k-means clustering.

2. Calculate the inner distances of all sample point pairs in *S*.

2.1. First, we define a graph *G *over all sample points by connecting points **p**_*i *_and **p**_*j *_in *S *if the line segment connecting **p**_*i *_and **p**_*j *_falls entirely within the object *O*, and an edge between **p**_*i *_and **p**_*j *_is added to the graph with its weight equal to the Euclidean distance ||**p**_*i *_- **p**_*j*_||.

2.2. Then, we compute the inner distances by applying a shortest path algorithm to the graph.

3. Build the signature of the shape *O *as the histogram of values of inner distances using 128 bins.

Our algorithm approximates the surface ∂*O *of *O *with a set of uniform sample points on ∂*O*, and the inner distances between each pair of sample points with the length of the shortest path through some other sample points. The implementation details of algorithm are presented next.

### Sampling points

The input shape of a molecule in the volumetric data is a point array. If all points of the boundary surface ∂*O *of the volumetric shape *O *are utilized for the final inner distance computation, it will increase the storage and computing costs of the shape inner distances. Therefore, we first sample points from the point array of the molecular surface ∂*O*. One issue of concern is the sample density. The more samples we take, the more accurately and precisely we can reconstruct the shape distribution. However, a large number of sample points increases the storage and computation costs of the inner distances, so there is an accuracy/time tradeoff in the choice of the number *m *of sample points. In our experiments, we have found that using *m *∈ [300, 1000] yields shape distributions with low enough variance and high enough resolution to be useful for our initial experiments.

A second issue is the sample method. We implement two sampling methods: random and uniform sampling. Random sampling method can not yield a good approximation using part of points. In this paper, we use Lloyd's algorithm of k-means clustering for obtaining uniform sampling points on a molecular surface. The uniform sample method consists of the following steps: 1) first, *m *random sample points are set as *m *clustering centers; 2) for each center, we cluster its neighborhood points; 3) each stage of Lloyd's algorithm moves every center point to the centroid of the cluster and then updates the cluster by recomputing the distance from each point to its nearest center; 4) these above steps are repeated until convergence; 5) finally, the point in each cluster, which is most nearest to the cluster center, is chosen as the final sample point. The C++ source code for k-means clustering can be found at: .

### Checking intersection

In the second step of IDSS, we check whether a line segment connecting two sample points falls entirely within the given shape *O*, which is called *inside visibility*. We check whether **p**_*i *_and **p**_*j *_are the inside visibility by computing the intersection between the boundary surface ∂*O *and the line segment **l **connecting **p**_*i *_and **p**_*j*_. Since ∂*O *is a point array or point cloud, this section will deal with the intersection between **l **and the point cloud surface ∂*O*. We improved our previous algorithm [56], called LPSI (Line and Point Sets Intersecting), for resolving the intersection problem. This algorithm is fast, robust and obtains the high accuracy without requiring a reconstruction of the underlying surface from point cloud. Our algorithm first detects whether an intersection has occurred between **l **and ∂*O*, and collects the inclusion points. Next we cluster the inclusion points. Finally the number of the resultant clusters is equal to the number of intersection points, which is used to judge the inside visibility.

We consider a cylinder around the line segment **l **with the radius *r*. To determine *r*, we need to obtain the density *ρ *of the point set ∂*O*, where *ρ *is the maximum size of a gap in ∂*O*. Suppose that *d *is the edge length of a voxel and it usually is set as a unit value, i.e. *d *≡ 1, then the longest distance in the neighborhood around a voxel is . Therefore, the density radius is chosen as *ρ *=  in this paper. Typically, we choose *r *= *ρ *=  as the radius of the cylinder for obtaining sufficient intersection points and less time. An intersection is reported if the cylinder contains some points of ∂*O*. We call these points inside the cylinder *inclusion points*.

After collecting the inclusion points, we then cluster them. Our cluster method maps 3D points into 1D parameter coordinates by projecting the inclusion points into the line segment **l**. Suppose that {**q**_*i*_} ⊆ ∂*O *is a set of inclusion points of **l **and ∂*O*. Firstly, we project each point **q**_*i *_onto **l**, and get one corresponding parameter *t*_*i *_∈ ℝ. We also obtain a set {*t*_*i*_} of parameters. Secondly, the set {*t*_*i*_} is sorted in increasing order. Here we suppose below that {*t*_*i*_} has already been sorted. Finally, we build the initial clusters by {*t*_*i*_} as described here. Starting from the minimal parameter of {*t*_*i*_}, a cluster *Q*_0_, which is a set of some inclusion points in {**q**_*i*_}, is built by comparing the distance of adjacent parameters. This cluster is terminated when the distance of two adjacent parameters is larger than a maximum bound (we typically choose 1.5 *d *as the bound). Then, starting from the terminated parameter, the next cluster *Q*_1 _is built repetitively. Clustering is terminated until the maximal parameter is reached. According to the number of initial clusters, we classify the intersection into three cases: According to the number of initial clusters, we classify the intersection into the following four cases.

**Case 1: **Containing only one intersection point (inside visibility).

**Case 2: **Containing two intersection points (either inside or outside visibility).

**Case 3: **Containing more than two intersection points (non-visibility).

For Case 1 that the number of intersection points is less than 2, **p**_*i *_and **p**_*j *_is inside visibility. For Case 3 the number of intersection points is more than 2, **p**_*i *_and **p**_*j *_is non-visibility.

For Case 2 that the number of intersection points is equal to 2, **p**_*i *_and **p**_*j *_are either inside or outside visibility. We collect inclusion points of **l **with *O *(not ∂*O*) and cluster inclusion points using the above strategy. If there is only one intersection point, **p**_*i *_and **p**_*j *_are inside visibility; otherwise, **p**_*i *_and **p**_*j *_are outside visibility.

After all pairs of sample points are checked for inside visibility, we define the graph *G *over all sample points by connecting points **p**_*i *_and **p**_*j *_and setting edge weight equal to the Euclidean distance ||**p**_*i *_- **p**_*j*_|| if **p**_*i *_and **p**_*j *_are inside visibility. An example is shown in Figure 4.

### Computing the shortest pathes

We estimate the inner distances between all sampling point pairs by computing their shortest path distances in the graph *G*. Algorithms for finding the shortest paths in graph are well known. Here we use Dijkstra's algorithm to compute the inner distance between sampling points in the graph *G*. Dijkstra's algorithm is a graph search algorithm that solves the single source shortest path problem for a graph. In order to implement Dijkstra's algorithm more efficiently, Fibonacci heap is used as a priority queue. We use the code package of Dijkstra's algorithm implemented by Tenenbaum et al. [57] (see ). In this paper we are interested in the inner distance between all pairs of sample points. The time complexity is *O*(*m*^3^) for *m *sample points.

### Building signatures

The inner distances reflect well the complex shape structure and articulated without explicitly decomposing shapes into parts. Now we convert a set of the inner distances defined on the boundary of the object to a shape signature. This is done in a similar manner as shape distribution in [7,29]. Given *m *sample points, the number of inner distances of the shape is at most *m*^2^/2. Specifically, we evaluate *m*^2^/2 inner distance values from the shape distribution and construct a histogram by counting how many values fall into each of *N*_*bin *_fixed sized bins. This vector with *N*_*bin *_entries is an expressive signature, as can be seen in Figure 1. Empirically, we have found that using *m *= 500 samples and *N*_*bin *_= 128 bins yields shape signatures with low enough variance and high enough resolution to be useful for our experiments.

### Similarity measurement

A signature of a shape is usually used as an index in a database of shapes and enables fast queries and retrieval. Hence, to achieve accurate results there is a need to define the similarity measurement between two shape signatures. Note that the shape signature of each molecule is represented by a 1D vector. There have been many standard ways of comparing two vectors investigated in [7]. These include *L*_*p *_(*p *= 1, 2,..., ∞) norms, the *χ*^2 ^measurement and Bhattacharyya distance. In fact, we have found that using different metrics on different signatures may affect lightly the query results. Although in our experiments we tested all different types of metrics for each signature when possible, we have found that the metrics such as *L*_1 _and *L*_2 _norms are simple and usually give better results. Assume that **I**_*A *_and **I**_*B *_represent signatures for two molecules *A *and *B*, respectively. The *L*_1 _norm, known as the Manhattan distance, between *A *and *B *is defined as

(2)

where **I**_*A*_(*i*) is the *i*th element of vector **I**_*A*_, similarly for **I**_*B*_(*i*). The *L*_2 _norm, known as the Euclidean distance, between *A *and *B *is defined as

(3)

## Authors' contributions

YL generated the original idea, executed the research, and wrote the manuscript. YF participated in the research. KR supervised the project and edited the paper. All authors read and approved the final manuscript.
